# Circulating MicroRNA Expression, Vitamin D, and Hypercortisolism as Predictors of Osteoporosis in Elderly Postmenopausal Women

**DOI:** 10.1155/2021/3719919

**Published:** 2021-12-13

**Authors:** Hadeel A. Al-Rawaf, Ahmad H. Alghadir, Sami A. Gabr

**Affiliations:** ^1^Department of Clinical Laboratory Sciences, College of Applied Medical Sciences, King Saud University, Riyadh, Saudi Arabia; ^2^Department of Rehabilitation Sciences, College of Applied Medical Sciences, King Saud University, Riyadh, Saudi Arabia

## Abstract

**Background:**

MicroRNAs (miRNA) identified as critical molecular regulators for bone development, function, and modeling/remodeling process and could be predictable for osteoporotic fractures in postmenopausal elderly women.

**Aim:**

The potential diagnostic role of circulating miRNAs, miR-148a and miR-122-5p, in the pathogenesis of osteoporosis and its association with bone markers, hypercortisolism, and vitamin D deficiency were explored in postmenopausal elderly women with osteoporosis.

**Methods:**

A total of 120 elderly women aged 50–80 years old were recruited in this study, of which only 100 eligible women with amenorrhea of at least 12 consecutive months or surgical menopause participated in this study. Based upon bone mineral density (BMD) measurements, the participants were classified according into two groups: normal (*n* = 45; *T* score of ≥-1.0) and osteoporosis (*n* = 55; *T* score: ≤-2.5). Circulating miRNAs, miR-148a and miR-122-5p, were estimated by real-time RT-PCR analysis. In addition, bone markers, hypercortisolism, and vitamin D deficiency were colorimetrically and ELISA immune assay estimated. The potential role of miR-148a, miR-122-5p, cortisol, and vitamin D in the diagnosis of osteoporosis was predicted using the analysis of the respective area under the receiver operating characteristic curve (AUC-ROC).

**Results:**

The expressed level of miR-148a significantly increased and miR-122-5p significantly decreased in the serum of osteoporotic patients compared to healthy controls. In addition, a significant increase in the levels of cortisol, s-BAP, and CTx and significant decrease in the levels of T-BMD, the levels of OC, and s-Ca were also identified. All parameters significantly correlated with fracture risk parameters; BMD, and *T* score lumbar spine (L2-L4). Thus, the data showed AUC cut off values (miR-148a; 0.876, miR-122-5p; 0.761) were best evaluated for clinical diagnosis of patients with osteoporosis and that AUC cut off values of 0.748 for cortisol and 0.635 for vitamin D were the best cut off values, respectively, reported for the prediction of osteoporosis clinical diagnosis.

**Conclusion:**

In this study, expressed miRNAs miR-148a and miR-122-5p and changes in the levels of both cortisol and vitamin D status are significantly associated with bone loss or osteoporosis. Thus, circulation miRNAs alone or in combination with cortisol and vitamin D status might be considered predictable biomarkers in the diagnosis or the pathogenesis of osteoporosis in elderly postmenopausal women; however, more studies are recommended.

## 1. Introduction

Bones are the most supporting component comprising the musculoskeletal system. It serves to support the body, assisting in movement, and protects vital organs as well as participates in producing blood cells, storing nutrients and minerals of the human bodies [1].

In normal biological processes, bone tissue is continuously controlled by hemostatic physiological modeled and remodeled processes to maintain a healthy bone matrix and to adapt to the change in environmental factors [1–3]. Thus, any disorder in these mechanisms significantly results in a reduction in bone mass with an increased risk for fractures with aging particularly, in postmenopausal women, impacting bone health [4, 5].

Osteoporosis is one of the most metabolic bone disease characterized by an imbalance in skeletal remodeling, resulting in an increase in the activity of osteoclast and/or a decrease in the numbers of osteoblasts. These hemostatic changes significantly lead to a decrease in bone strength, mass, bone quality, bone density, and bone geometry with lower bone mineral density (BMD) of ≤2.5 standard deviations below peak bone mass which significantly increases the susceptibility to severe bone fracture especially around age 30 [6–9]. BMD considered one of the most important measurements for predicting osteoporotic fractures among older ages [10, 11]. However, other several physiological factors, such as hormones, vitamin D deficiency, cytokines, and mechanical stimulation, significantly affect resorption and formation of bone markers and mediate the complex regulation processes of bone homeostasis [12–25].These factors could interfere with other factors such as exercise, age, sex, and diet with the pathogenesis and severity of osteoporosis [26].

In addition, many studies reported that proinflammatory cytokines and their related genes such as galectin, NLRP3, and IL-6 have a significant pivotal role in the destruction of bones and severity of osteoporosis. More genes like interleukin 6 (IL-6), vitamin D receptor (VDR), estrogen receptor (ESR), and calcitonin receptor (CTR) as well others were suggested to be associated with osteoporosis and bone density [27–34]. Previous clinical studies found that IL-6 and other inflammatory cytokines accelerate bone destruction and osteoporosis via upregulation of the expression and the signal transduction of RANKL and on osteoblasts, increasing vertebral fractures and the severity of bone loss or osteoporosis [35–38].

Pathophysiological hypotheses reported that proinflammatory cytokines like IL-6 along with matrix GLA protein and growth factors are associated with bone turnover and in turn have a role in bone loss or osteoporosis in patients with ischemic cardiovascular diseases [39]. Additionally, NLRP3 inflammasome was activated as inflammatory promoters in patients with osteoporosis which was significantly suppressed by melatonin use in patients with postmenopausal osteoporosis [40, 41].

As general, galectins as carbohydrate-binding proteins interfere in many physiological processes particularly inflammatory and immune response showed to be associated with activation of other proinflammatory markers such as NLRP3 inflammasome and aid in the progression of osteoporosis [42, 43]. NLRP3 and galectins showed to be significantly elevated in the serum and saliva of patients with periodontitis, confirming the association of with a multifactorial inflammatory disease [33, 34]. In addition, clinical studies reported that asymptomatic hypercortisolism is associated with osteoporotic fractures and that subclinical hypercortisolism (SH) is among the causes with bone fragility particularly those with slightly reduced BMD and vertebral fractures [44].

In addition, non-coding short RNA molecules (18-25 nucleotides) showed to be associated with many diseases [26–28] including the bone loss or osteoporosis [29–32].

Several environmental and genetic factors significantly interfere with the incidence and the pathogenesis of osteoporosis [14]. Epigenetic mechanisms including microRNA expression is shown to be associated as posttranscriptional regulators of gene expression [45, 46]. The prominent role of miRNAs was demonstrated in many physiological processes proceeding in the pathogenesis of a number of diseases [47]. Remarkable stability, protection from degradation, tissue specificity, and easy accessibility of circulating miRNAs molecules support its use as biomarkers for early diagnosis for diseases and monitoring of treatment [48–52]. Previous studies reported that extracellular miRNAs represent as a cellular waste product and might be required in intercellular communication [48, 49]. In bone formation, miRNAs are identified to be linked with the differentiation, proliferation, and function of both osteoblasts and osteoclasts [53]. Also, bone homeostasis was regulated by the miRNAs via direct or indirect impact on the expression of various transcription factors and cytokines required in the process of bone resorption and bone formation [53–55]. In addition, the functional role of microRNAs in bone regulation suggests their vital role as biomarkers for pathological changes associated with osteoporosis [56]. This support the role of miRNAs in many bone diseases which is not fully studied [53, 56–64]; however, their biological role in the pathogenesis of bone and its association with other physiological parameters like hypercortisolism and vitamin D deficiency are still unclear. From the previously mentioned studies [14, 45–55], it was reported that in postmenopausal patients, more physiological parameters like hypercortisolism, vitamin D deficiency, and circulating miRNAs were associated with osteoporotic fractures. However, in postmenopausal women with osteoporosis, there are little or no intercorrelations were fully studied between expressed circulating miRNAs with hypercortisolism, vitamin D deficiency, and physiological bone markers. Thus, in this study, the potential diagnostic role of circulating miRNAs, miR-148a and miR-122-5p, in the pathogenesis of osteoporosis and its association with bone markers, hypercortisolism, and vitamin D deficiency were explored in postmenopausal elderly women with osteoporosis.

## 2. Materials and Methods

### 2.1. Subjects

A total of 120 elderly women aged 50–80 years old were recruited in this study, of which only 100 eligible women with amenorrhea of at least 12 consecutive months or surgical menopause participated in this study. Based upon bone mineral density (BMD) measurements, the participants were classified according into two groups: normal (*n* = 45; *T* score of ≥ -1.0) and osteoporosis (*n* = 55; *T* score: ≤-2.5). Study exclusion criteria included women with severe disease complications such as diabetes mellitus, chronic liver diseases, rheumatoid arthritis or collagen, endocrine disorders which affect bone mass, hyperthyroidism, spondylitis, systemic lupus erythematosus, connective tissue disease, metabolic and endocrine diseases, bone tumours, or who received corticosteroid therapy, hormone replacement therapy, stress hormones, or the miRNA measurements, and bisphosphonates treatment that could interfere with BMD measurements. In addition, patients with low physical activity, prevalent fractures, or with parental history of bone disorders were excluded either from the current study.

The study protocol was reviewed according to the ethical guidelines of the 1975 Declaration of Helsinki and approved by the ethical committee of Rehabilitation Research Chair (RRC), King Saud University, Kingdom of Saudi Arabia, under file number (ID: RRC-2019-096), and signed informed consent forms were received from all subjects prior data collection.

### 2.2. Diet and Duration of Sun Exposure

During the entire period of data collection, all participants were informed not to change their normal eating habits and to record accurately the amount, type of food, and fluid consumed. Then, dietary information was obtained from food diaries of each participant or by extensive dietary interviews and significantly referred according to reference dietary intakes previously reported [65, 66]. In addition, daily sun exposure was estimated by the number of weeks spent in the sun by each student and divided by seven to estimate the average number of minutes per day the participants; normal and osteoporosis were exposed to sunlight [65, 66].

### 2.3. Blood Sampling

Following an overnight fast, blood samples were obtained from all participants at 9:00–11:30 a.m. to avoid probable diurnal influence. The proposed time for blood collection was selected for control of the circadian hormonal range, as previously reported in the procedures of other studies [67].

### 2.4. Assessment of BMD Measurements

All participants were subjected to perform BMD measurements of the lumbar spine, total hip, femoral neck and 1/3 radius by using dual-energy X-ray absorptiometry (Discovery, Hologic, Waltham, MA) as previously reported [68]. Osteoporosis was significantly defined according to the criteria of World Health Organisation (WHO) [69–71]. According to these diagnostic criteria, subjects were classified as osteoporosis (DXA *T* scores of ≤ −2.5 standard deviation (SD)), osteopenia (*T* scores of −1.0 SD to −2.5 SD), and normal [*T* scores of ≥−1 SD], respectively [68–71].

### 2.5. Assessment of Bone Markers

Serum levels of calcium, bone alkaline phosphatase (sBAP), osteocalcin (OC), and C-telopeptide (CTx) were estimated in all participants as previously reported (63, 67, 68, 69, and 16). Serum calcium and BAP concentrations were estimated by a Cobas Integra colorimetric analyzer with the aid of commercially available immunoassay ELISA kits (Hoffman-LaRoche Ltd., Basel, Switzerland) for calcium and the immunoenzymetric assay kits (MicroVue BAP, Quidel Corporation, San Diego, CA) for serum BAP concentrations [69–71].

In addition, serum levels of OC and CTx were determined using enzyme immunoassay analysis [72, 73]. The levels were measured with ELISA kits (the MicroVue Osteocalcin enzyme immunoassay, Quidel Corporation, San Diego, CA) for OC and enzyme immunoassay kits (Serum CrossLaps One Step ELISA, Osteometer BioTech, Herlev, Denmark)) for serum CTx, respectively [72, 73].

### 2.6. Assessment of Vitamin 25(OH)D and Cortisol

Serum concentrations of vitamin 25(OH)D and cortisol were estimated in all subjects as previously mentioned in the literature [74, 75]. In this experiment, immunoassay ELISA kits (IDS, Tyne & Wear, UK) and (ELISA, Diagnostics Biochem. Canada Inc.) were used to estimate the levels of vitamin 25(OH)D and cortisol concentrations, respectively, in the serum of all participants.

### 2.7. Real-Time RT-PCR Analysis of Circulating miRNAs and Apoptotic Genes

#### 2.7.1. Extraction of RNA and Synthesis of *cDNA*

For each participant, the miRNease isolation kit (Qiagen, Hilden, Germany) was used to extract total RNA from serum samples. A reverse-transcription polymerase chain reaction (RTPCR) was applied to analyze total RNA in all serum samples. Then, a complementary DNA (c-DNA) was generated using reverse transcription miScriptII RT kits (Qiagen), and the levels of miRNAs were evaluated by optical density [76, 77].

#### 2.7.2. Real-Time RT-PCR Analysis

The primers of circulating miRNAs miR-148a and miR-122-5p (Applied Biosystems, Foster City, CA, U.S.A.) were used to screen the expression of miRNAs in the plasma of all participants by using a quantitative real-time RT-PCR [78]. The average copy number of the resultant PCR components was normalized according to the GAPDH gene which is used as an internal housekeeping gene [79, 80]. In the PCR process, templets of respective cDNA were subjected to four thermal phases: primary denaturation phase (I) (at 94°C for 2 minutes), denaturation phase (II) (at 94°C for 30 seconds), annealing phase (III) (at 59°C for 30 seconds), and amplification phase (IV) (at 72°C for 30 seconds). The PCR phases (II to IV) proceed for 45 cycles and all reactions were measured in a triplicated manner [81].

#### 2.7.3. Statistical Analysis

Power calculations of the selected sample size of 100 subjects showed to give an estimated power of 96% and a significance level of 0.05 with an expected frequency of 9.6%.

An SPSS statistical program (SPSS, IBM Statistics V.17) was used to analyze all data produced in this study. The data of continuous variables are expressed as mean ± SD. The frequency differences between the groups were analyzed by using a nonparametric test (Mann–Whitney-Wilcoxon test) and the *χ*^2^ test, respectively. In all groups, two independent sample *t*-tests were used for comparison between the studied variables such as osteoporosis (dependent variable), expression levels of miRNAs, bone markers, vitamin 25(OH)D, and serum cortisol levels (independent variables). In addition, multiple stepwise regressions and Pearson's correlations analysis were used to estimate the associations between bone loss (osteoporosis) and the studied independent variables in control subjects and in postmenopausal women with osteoporosis. The susceptibility and sensitivity of cortisol, vitamin D, miR-148a, and miR-122-5p for osteoporosis diagnosis at baseline expression were determined using the area under the receiver operating characteristic (ROC) curve as previously reported [81]. All tests were two-tailed; because of multiple assessments, results were only considered statistically significant at a value of *p* < 0.05.

## 3. Results

In this study, a total of 100 eligible women with amenorrhea of at least 12 consecutive months participated in this study. Only 55% of the study population reported osteoporosis with *T* score (≤-2.5) and 45% of the population had normal *T* score (≥-1.0), respectively (Table 1). Fracture risk parameters, T-BMD, BMD-femoral neck, BMD-total hip, BMD-lumbar spine, BMD 1/3, and *T* score, were significantly decreased (*p* = 0.001) in osteoporotic women compared to healthy controls, whereas BMD (*z* and *T* scores) at FN and at LS were significantly different (*p* = 0.001) (Table 1).

In addition, deficient in dietary vitamin D and Ca intake were significantly reported in osteoporotic women (*p* = 0.001) compared to healthy controls.

**Figure 1:**
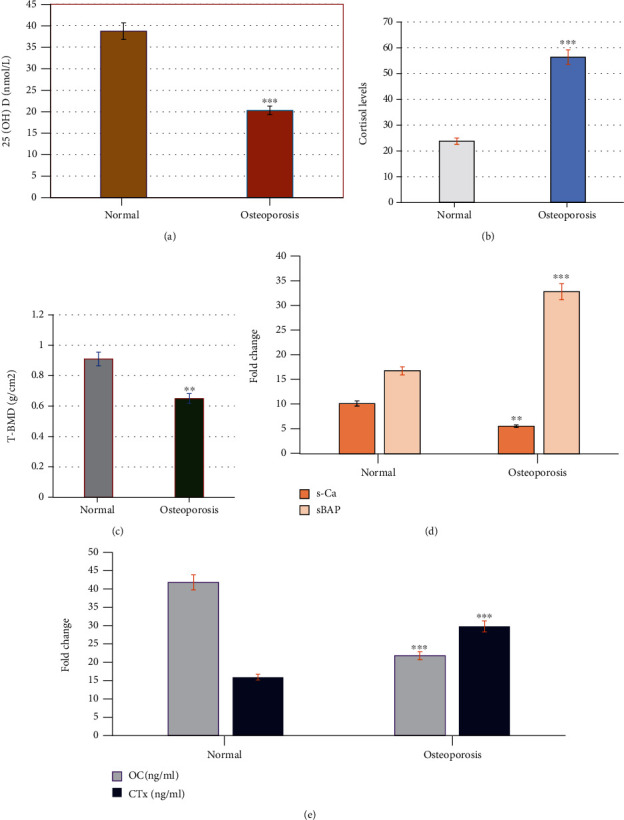
Changes in vitamin D and cortisol (a, b) total bone mineral density and bone markers; OC, CTx; sBAP and s-Ca the levels (c–e) in healthy control and in postmenopausal women with osteoporosis. The results showed significant decrease (*p* = 0.001) in the levels of vitamin D (a) and increase (*p* = 0.001) in the levels of cortisol (b) in osteoporotic postmenopausal women compared to healthy controls. In addition, significant decrease (*p* = 0.001) in the T-BMD, the levels of OC, s-Ca, and increased levels of sBAP, CTx (*p* = 0.001), respectively, in osteoporotic postmenopausal women compared to healthy controls (c–e). ^∗∗^*p* ≤ 0.01, ^∗∗∗^*p* ≤ 0.001. OC: osteocalcin; CTx: collagen type I c-telopeptide; sBAP: serum bone alkaline phosphatase; s-Ca: serum calcium.

In this study, the effect of vitamin D and cortisol levels on the bone loss was estimated in healthy and osteoporotic women (Figure 1). A significant decrease (*p* = 0.001) in the levels of vitamin D (a) and increase (*p* = 0.001) in the levels of cortisol (b) were reported in osteoporotic postmenopausal women compared to healthy controls. In addition, a significant decrease (*p* = 0.001) in the T-BMD, the levels of OC and s-Ca, and increased levels of s-BAP and CTx (*p* = 0.001), respectively, in osteoporotic postmenopausal women compared to healthy controls (Figures 1(c)–1(e)).

**Figure 2:**
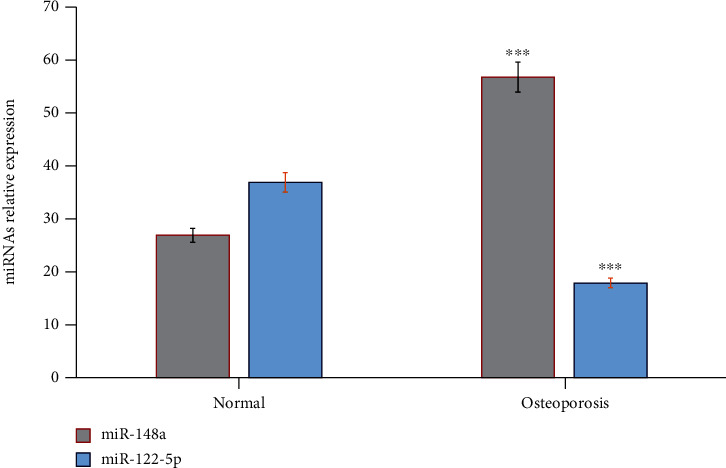
MicroRNAs' differential expression profile in healthy control and in postmenopausal women with osteoporosis. The results showed that the relative expression of miR-148a significantly increased (*p* = 0.001) and miR-122-5p significantly reduced (*p* = 0.001) in postmenopausal women with osteoporosis compared to healthy controls. ^a^*p* ≤ 0.01, ^b^*p* ≤ 0.001.

Molecular changes associated with osteoporosis in postmenopausal women were also studied (Figure 2). MicroRNAs' differential expression profile was estimated in healthy control and in postmenopausal women with osteoporosis. The results showed that the relative expression of miR-148a significantly increased (*p* = 0.001), and miR-122-5p significantly reduced (*p* = 0.001) in postmenopausal women with osteoporosis compared to healthy controls.

In women with osteoporosis, the association of fracture risk parameters; T-BMD and *T* score lumbar spine (L2-L4) with cofounders were evaluated (Table 2). Both T-BMD and *T* score correlated negatively with the change in the levels of vitamin-25 (OH)D, cortisol, OC, and s-Ca and positively correlated with CTx and s-BAP, respectively, as shown in Table 2.

In addition, the cellular expression microRNAs; miR-148a and miR-122-5p were significantly associated (*p* = 0.001) with the levels of vitamin-25 (OH)D, cortisol, and bone markers: OC, CTx, s-Ca, and s-BAP. The expression of miR-148a and miR-122-5p correlated positively with OC, CTx, s-Ca, and s-BAP and negatively with BMD, and *T* score lumbar spine (L2-L4), the levels of vitamin-25 (OH)D, cortisol, respectively, as shown in Table 3.

Diagnostic values for cortisol, vitamin D, miR-148a, and miR-122-5p in clinical samples for osteoporosis patients were determined using the area under the receiver operating characteristic (ROC) curve (Table 4). There were acceptable diagnostic values for the levels of miR-148a (AUC = 0.876, *p* = 0.01) and miR-122-5p (AUC = 0.761, *p* = 0.05) in clinical samples for osteoporosis patients (Table 4). The sensitivity and specificity for miR-148a in clinical samples were 91.5% and 89.3% compared to that of miR-122-5p, 82.9% and 86.7%, respectively. In addition, there was a much stronger diagnostic value for osteoporosis (AUC = 0.820, *P* = 0.001) reported when both microRNAs combined together than either separately (Table 4).

Similarly, there were acceptable diagnostic values for the levels of cortisol (AUC = 0.748, *p* = 0.01) and vitamin D (AUC = 0.635, *p* = 0.01) in clinical samples for osteoporosis patients (Table 4). The sensitivity and specificity for cortisol in clinical samples were 89.8% and 85.2% compared to that of vitamin D 85.8% and 84.6%, respectively. In addition, there was a much stronger diagnostic value for osteoporosis (AUC = 0.789, *p* = 0.001) reported when both cortisol and vitamin D combined together than either separately (Table 4). The results suggest that both microRNAs, miR-122-5p and miR-4516, along with cortisol, and vitamin D status could be used together to increase diagnostic value for osteoporosis.

## 4. Discussion

Osteoporosis comprises a major public health problem characterized by a reduction in bone mass and deterioration in bone microarchitecture with lower bone mineral density (BMD) values (*T* score; 2.5 s.d or more) at the spine or hip regions which significantly below the average calculated values for healthy young women [82].

In this study, osteoporosis was estimated in 55% (*n* = 55) of the study population with *T* score (≤-2.5). Fracture risk parameters: T-BMD, BMD-femoral neck, BMD-total hip, BMD-lumbar spine, BMD 1/3, and *T* score were significantly decreased (*p* = 0.001) in osteoporotic women compared to healthy controls (*T* score; ≥-1.0).

An increase in osteoporosis was detected among postmenopausal women which significantly depend mainly upon the type of clinical techniques for BMD measurement [81–83]. In these studies, although, there was significant variability in detecting osteoporosis between used techniques such as QCT and DXA; osteoporosis still present in higher values among postmenopausal women.

Our results were significantly supported by an abnormal change in the levels of both resorption and formation-related bone markers. In osteoporotic postmenopausal women, the levels of OC and s-Ca significantly decreased and the levels of s-BAP and CTx significantly increased compared to healthy control women.

Previously, it was suggested that bone turnover markers such as bone resorption and bone formation markers can be used alone or in association with other bone parameters for assessing bone loss or osteoporosis in aged menopause women [84, 85].

After menopause, the role of endogenous glucocorticoids and deficient in vitamin D in physiological bone loss remain to be explored. Subclinical hypercortisolism [86, 87] and lower vitamin levels were reported among older patients with established osteoporosis [88, 89].

Thus, in the current study, a significant decrease (*p* = 0.001) in the levels of vitamin D with an increase (*p* = 0.001) in the levels of cortisol was reported in osteoporotic postmenopausal women compared to healthy controls.

Our results like others suggest that the physiological changes in cortisol levels significantly associated with impairment of bone mass and bone quality in physiological menopause. Thus, postulate that the parameters of the hypothalamic-pituitary-adrenal (HPA) axis function may contribute to postmenopausal bone health [89–92]. The prevalence of subclinical hypercortisolism was reported in about 10% of outpatients with established osteoporosis. Thus, the presence of hypercortisolism has to be taken into account when evaluating patients with unexplainable established osteoporosis [93]. In addition, adequate levels of serum vitamin D are required to protect against bone fracture [94, 95] and that the complications of vitamin D deficiency particularly the vitamin D status and mutations in the vitamin D receptor markedly contribute to bone health among elderly women [94, 96–98].

In this study, compared to controls, T-BMD and *T* score lumbar spine (L2-L4) as fracture risk parameters in osteoporotic patients correlated negatively with deficient vitamin-25 (OH)D, higher cortisol levels, and lower OC and s-Ca levels and positively correlated with higher levels of both CTx and s-BAP. It was reported previously that the deficiency in vitamin D levels was related to several factors particularly disturbance in the synthesis of physiological hormones which affects the production of the active forms of vitamin D [99–101].

Similarly, 25(OH)D and bone turnover markers (BTMs) shown to be associated with the incidence of hip fractures in older adults [102–110].Thus, a deficiency in serum 25(OH)D levels considered one of the most risk factors for the incidence of hip fractures in older women [102].

In addition, several studies investigated the unrecognized risk factors for bone loss [13, 110–113] especially cortisol. Like our results, others reported that excessive production of cortisol can lead to diminished bone metabolism and altered bone architecture [114], whereas a dysregulation of the hypothalamic-pituitary-adrenal (HPA) system significantly leads to secretions of cortisol in higher concentrations in the serum cortisol of osteoporotic patients which in turn is associated with a decrease in bone formation and increase in bone resorption with an overall decline in bone mineral content [13, 103–115].

Additionally, molecular-based studies represent microRNAs (miRNA) as critical molecular regulators that are involved in the bone remodeling processes, whereas several miRNAs showed to participate in several mechanisms especially, bone development, function, modeling, and remodeling process [101–104]. In addition, it plays a potential role in the characterization of the phenotype of bone cells (osteocytes, osteoblasts, and osteoclasts) [105, 106]. Thus, miRNAs showed to be involved in bone diseases [107].

In this study, the potential roles of miRNAs as molecular markers in the pathogenesis and early diagnosis of osteoporosis were estimated in osteoporotic patients by using a quantitative real-time RT-PCR. The results showed a significant increase in baseline serum miR-148a and a decrease in the levels of miR-122-5p in osteoporotic patients compared to healthy controls, respectively. The cellular expression of microRNAs miR-148a and miR-122-5p significantly correlated with risk parameters: T-BMD and *T* score lumbar spine (L2-L4), the levels of vitamin-25 (OH)D, cortisol, and bone markers OC, CTx, s-Ca, and s-BAP. The expression of miR-148a and miR-122-5p correlated positively with OC, CTx, s-Ca, and s-BAP and negatively with T-BMD and *T* score lumbar spine (L2-L4), vitamin-25 (OH)D, and cortisol, respectively.

Recently, the increased levels of miR-148a significantly correlated with the main parameters of bone metabolism: bone mineral density (BMD) total body (TB) values, calcium, osteocalcin, bone alkaline phosphatase (sBAP), and vitamin-25 (OH)D (VitD) [116]. These data significantly assigned the potential role of miR-148a in both bone metabolism alterations and remodeling.

Also, previous studies reported the increase of miR-148a in plasma of osteoporotic (OP) postmenopausal women [117] and in sera of those with reported hip fractures [118]. Thus, the circulating expression of miR-148a signature suggested a potential for bone remodeling and could be considered as a potential biomarker of bone diseases [13, 110–113].

MicroRNAs are expressed in cells and significantly reported as critical molecular regulators capable of modifying the expression of genes at a posttranscriptional level [13, 114]. This proceeds via inhibiting the translation of particular mRNAs or inducing specific mRNA degradation [115–117]. Consistent with our results, hsa-miR-122-5p was significantly downregulated in serum samples from osteoporosis patients compared to healthy nonosteoporosis subjects. These confirmed that the expressed microRNAs; miR-148a, miR-122-5p were associated with a fragility fracture and the low bone mineral density measured by T-BMD, and T-score lumbar spine (L2-L4 in osteoporosis patients [118, 119].

In the current study, ROC curve analysis showed that miR-148a, miR-122-5p along with both cortisol and vitamin 25 (OH)D levels could be identified as strong diagnostic osteoporotic biomarkers. The results are in line with previously reported studies that confirmed cellular expressional changes in the levels of both miR-148a, miR-122-5p in elderly women with osteoporosis [13, 113–119]. The expression of miR-122-5p is differentially down-regulated and miR-148a is differentially upregulated in the serum of osteoporotic elderly women [13, 112–120]. In addition, cortisol significantly increased [97, 98, 113, 115–117] and vitamin 25 (OH)D levels significantly reduced [103, 106, 107, 111] in osteoporotic women and clinically associated with bone fractures and low bone mineral density in osteoporosis patients.

Finally, although BMD measurement is gold standard for diagnosis of osteoporosis/osteopenia, the results obtained signify circulating miR-148a, miR-122-5p along with cortisol, and vitamin D status as potential diagnostic biomarkers for osteoporosis. Thus, these markers might be useful in cases where DXA analysis might be severe or unsuitable for application such as pregnancy.

## 5. Conclusion

In this study, expressed miRNAs miR-148a and miR-122-5p and changes in the levels of both cortisol and vitamin D status significantly associated with bone loss or osteoporosis. Thus, circulation miRNAs alone or in combination with cortisol and vitamin D status might be considered as predictable biomarkers in diagnosis or the pathogenesis of osteoporosis in elderly postmenopausal women, however more studies are recommended.

## Figures and Tables

**Table 1:** The demographics and baseline bone parameters of the participants.

Variables	Normal group	Osteoporosis group
*N*	45 (45%)	55 (55%)
Age (years)	62.6 ± 4.1	63.9 ± 3.2
BMI (kg/m^2^)	24.9 ± 3.4	22.5 ± 2.6^b^
Waist (cm)	96.8 ± 9.3	79.2 ± 6.3^b^
Hips (cm)	93.7 ± 6.4	89.8 ± 8.3^b^
WHR	1.034 ± 0.13	0.882 ± 0.09^b^
Years of menopause	7.5 ± 1.5	12.5 ± 2.0^b^
BMD femoral neck (FN; g/cm^2^)	0.751 ± 0.61	0.610 ± 0.39^b^
*T* score for FN	0.38 ± 0.56	−2.6 ± 0.75^b^
*Z* score for FN	1.3 ± 0.46	−1.42 ± 0.82^b^
BMD lumbar spine (LS; g/cm^2^)	0.985 ± 0.86	0.692 ± 0.58^a^
*T* score for LS (L2-L4)	0.71 ± 1.3	−2.8 ± 0.98^b^
*Z* score for LS	0.48 ± 1.1	−3.1 ± 0.98^b^
BMD total hip (g/cm^2^)	0.865 ± 0.812	0.715 ± 0.59^b^
BMD 1/3 radius (g/cm^2^)	0.68 ± 0.23	0.51 ± 0.48^b^
Total BMD (g/cm^2^)	0.91 ± 0.08	0.65 ± 0.09^b^
Sun exposure (min/d)	125 ± 15.8	80 ± 12.7^b^
Dietary vitamin D intake (IU/d)	215 ± 22.5	118 ± 8.3^b^
Dietary Ca intake (mg/d)	1350 ± 148	789 ± 31.5^b^

Values are expressed as mean ± SD; significance at *p* < 0.05. ^a^*p* < 0.01, ^b^*p* < 0.001. BMD: bone mineral density; BMI: body mass index; WHR: waist-to-hip ratio.

**Table 2:** Correlations between bone markers, cortisol, vitamin D levels, miRNAs, and fracture risk parameters in postmenopausal women with osteoporosis.

Studied parameters	Fracture parameters	
	Total BMD (g/cm^2^)	*T* score lumbar spine (L2-L4)
OC (ng/ml)	-0.27^a^	-0.36^b^
CTx (ng/ml)	0.23^a^	0.14^b^
s-Ca	-0.24^a^	-0.26^b^
s-BAP	0.38^a^	0.42^b^
Vitamin-25 (OH)D (nmol/l)	-0.31^a^	-0.42^b^
Cortisol	-0.18^a^	-0.21^b^

Data are presented as Pearson's (*R*) coefficients adjusting for variables identified as cofounders in univariate analyses. Significance at *p* < 0.05. ^a^*p* < 0.01, ^b^*p* < 0.001. BMD: bone mineral density; OC: osteocalcin; CTx: collagen type I c-telopeptide; sBAP: serum bone alkaline phosphatase; s-Ca: serum calcium.

**Table 3:** Correlations between bone markers, cortisol, vitamin D levels, and miRNAs in postmenopausal women with osteoporosis.

Studied parameters	miRNA expression	
	miR-148a	miR-122-5p
OC (ng/ml)	0.76 ^b^	0.64 ^b^
CTx (ng/ml)	0.42 ^b^	0.31 ^b^
s-Ca	0.58 ^a^	0.69 ^b^
s-BAP	0.18^a^	0.23 ^b^
Cortisol	-0.37^b^	-0.49 ^b^
Vitamin-25 (OH)D (nmol/l)	-0.17^a^	-0.39 ^b^
Total BMD (g/cm^2^)	-0.51^a^	-0.24 ^a^
*T* score lumbar spine (L2-L4)	-0.63 ^b^	-0.27 ^b^

Data are presented as Pearson's (*R*) coefficients adjusting for variables identified as cofounders in univariate analyses. Significance at *p* < 0.05. ^a^*p* < 0.01, ^b^*p* < 0.001. OC: osteocalcin; CTx: collagen type I c-telopeptide; sBAP: serum bone alkaline phosphatase; s-Ca: serum calcium.

**Table 4:** Diagnostic value of mi-R-148a, miR-122-5p, cortisol, and vitamin D for osteoporosis (*n* = 55).

miRNAs	AUC for diagnosis of osteoporosis (Ops)		
	Area (95% CI)	Sensitivity	Specificity
mi-R-122-5p	0.761^a^ (0.686-0.896)	82.9	86.7
miR-148a	0.876^b^ (0.798-0.965)	91.5	89.3
mi-R-148a+miR-122-5p	0.820^c^ (0.720-0.910)	94.2	89.6
Cortisol	0.748^b^ (0.650-0.875)	89.8	85.2
Vitamin D	0.635^b^ (0.580-0.785)	85.8	84.6
Cortisol+vitamin D.	0.789^c^ (0.650-0.896)	90.8	87.3

AUC: area under the curve; CI: confidence interval. ^a^*p* < 0.05. ^b^*p* < 0.01. ^c^*p* < 0.001.

## Data Availability

All data generated or analyzed during this study are presented in the manuscript. Please contact the corresponding author for access to data presented in this study.
